# Executive summary: British Society for Rheumatology guideline on management of adult and juvenile onset Sjögren disease

**DOI:** 10.1093/rheumatology/keae218

**Published:** 2024-05-24

**Authors:** Elizabeth J Price, Stuart Benjamin, Michele Bombardieri, Simon Bowman, Sara Carty, Coziana Ciurtin, Bridget Crampton, Annabel Dawson, Benjamin A Fisher, Ian Giles, Peter Glennon, Monica Gupta, Katie L Hackett, Genevieve Larkin, Wan-Fai Ng, Athimalaipet V Ramanan, Saad Rassam, Saaeha Rauz, Guy Smith, Nurhan Sutcliffe, Anwar Tappuni, Stephen B Walsh

**Affiliations:** Department of Rheumatology, Great Western Hospital NHS Foundation Trust, Swindon, UK; The Academy Library and Information Service, Great Western Hospital NHS Foundation Trust, Swindon, UK; Department of Rheumatology, Barts and The London School of Medicine and Dentistry, Barts Health NHS Trust, London, UK; Centre for Experimental Medicine and Rheumatology, The William Harvey Research Institute, Queen Mary University of London, London, UK; Department of Rheumatology, Milton Keynes University Hospital, Milton Keynes, UK; Department of Rheumatology, University Hospitals Birmingham NHSFT, Birmingham, UK; Institute of Inflammation and Ageing, University of Birmingham, Birmingham, UK; Department of Rheumatology, Great Western Hospital NHS Foundation Trust, Swindon, UK; Centre for Rheumatology, Division of Medicine, University College London, London, UK; Patient Representative, Sjogren’s UK helpline lead, Sjogren’s UK (British Sjögren’s Syndrome Association), Birmingham, UK; Patient Representative, Sjogren’s UK (British Sjögren’s Syndrome Association), Birmingham, UK; Rheumatology Research Group, Institute of Inflammation and Ageing, College of Medical and Dental Sciences, University of Birmingham, Birmingham, UK; National Institute for Health Research (NIHR) Birmingham Biomedical Research Centre and Department of Rheumatology, University Hospitals Birmingham NHS Foundation Trust, Birmingham, UK; Centre for Rheumatology, Division of Medicine, University College London, London, UK; General Practice, NHS Staffordshire & Stoke on Trent ICB, Stafford, UK; Department of Rheumatology, Gartnavel General Hospital, Glasgow, UK; Department of Social Work, Education and Community Wellbeing, Northumbria University, Newcastle upon Tyne, UK; Department of Ophthalmology, Kings College Hospital, London, UK; Translational and Clinical Research Institute & Newcastle NIHR Biomedical Research Centre, Newcastle University, Newcastle upon Tyne, UK; Department of Rheumatology, Newcastle upon Tyne NHS Foundation Trust, Newcastle upon Tyne, UK; Paediatric Rheumatology, Bristol Royal Hospital for Children, Bristol, UK; Translational Health Sciences, University of Bristol, Bristol, UK; Haematology and Haemato-Oncology, KIMS Hospital, Maidstone, Kent, UK; Ophthalmology, Institute of Inflammation and Ageing, University of Birmingham, Birmingham, UK; Birmingham and Midland Eye Centre, Sandwell and West Birmingham NHS Trust, Birmingham, UK; Department of Ophthalmology, Great Western Hospital NHS Foundation Trust, Swindon, UK; Department of Rheumatology, Barts Health NHS Trust, London, UK; Institute of Dentistry, Queen Mary University of London, London, UK; London Tubular Centre, University College London, London, UK

## Abstract

**Graphical abstract:**
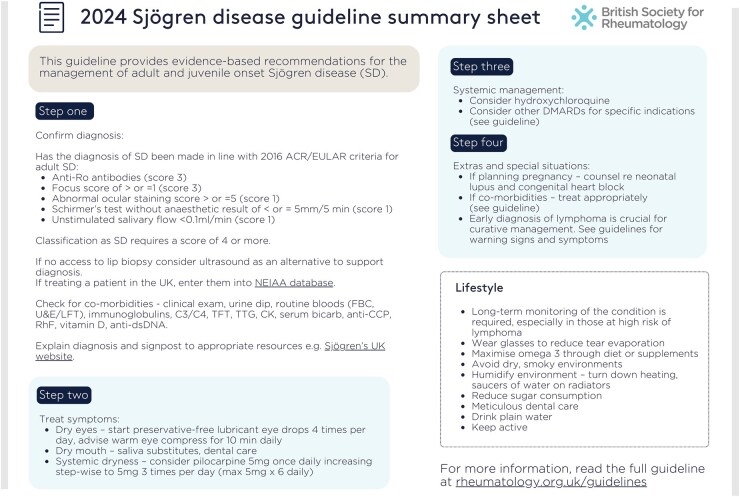


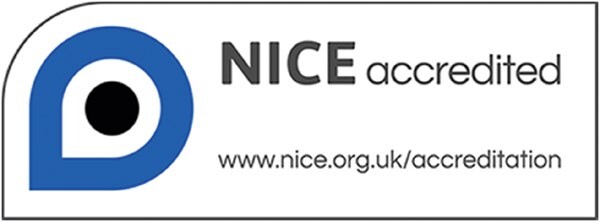


NICE has accredited the process used by BSR to create its clinical guidelines. The term began on 27 February 2012 and the current renewed accreditation is valid until 31 December 2023. More information on accreditation can be viewed at www.nice.org.uk/accreditation.

This is the executive summary of ‘British Society for Rheumatology guideline on management of adult and juvenile onset Sjögren disease.’ For the full guideline, please see https://doi.org/10.1093/rheumatology/keae152.

## Background and rationale for guideline development

Sjögren disease (SD) is a chronic, auto-immune disease of unknown aetiology with significant impact on quality of life (QoL) [1]. Although dryness (sicca) of the eyes and mouth are the classically described features, dryness of other mucosal surfaces and systemic manifestations are common. The key management aim should be to empower the individuals to manage their condition; conserving, replacing and stimulating secretions; preventing damage and suppressing systemic disease activity.

This guideline builds on, and widens the recommendations developed for the first guideline published in 2017 [2]. We have included advice on the management of children and adolescents where appropriate to provide a comprehensive guideline for UK-based rheumatology teams.

## Guideline development

This guideline was developed in line with the British Society for Rheumatology (BSR) Creating Guidelines protocol. The working group agreed the guideline scope and identified 19 key questions [3]. Using these key questions, a literature search was undertaken (Supplementary Data S1, available at *Rheumatology* online), eligible papers were reviewed, draft recommendations developed and the GRADE process followed to summarize the quality of evidence as high (A), moderate (B) or low/very low (C) [4].

The content, wording, strength of recommendation (strong = 1, conditional = 2) and Strength of Agreement (SoA) were determined by the working group responses. Only recommendations with a SoA >80% were included.

## Key questions identified in the scope

### 1. In people suspected of SD, what is the diagnostic accuracy of antinuclear antibodies (ANA), extractable nuclear antigens (ENA) and other novel antigen testing?

Studies [5–10] (see Table 1) estimate the sensitivity of ANA as 58–85% and specificity as 50–97%. ANA is commonly used as a screening antibody but because of its frequency and low specificity, should not be measured in the absence of clinical indicators.

**Table 1.** Summary of evidence on diagnostic accuracy of antinuclear antibodies (ANA) in SD and various CTDs

Study	Population	Diagnosis	Index tests	Ref standard	Comments	Sensitivity % & Specificity % (95% CI) of ENA in CTD	AURC
Jeong *et al.* 2018 [5]	N = 1115, suspected of AARD; Of whom 19 were diagnosed with SS	Various AARDs	ANA—indirect immunofluorescence	Expert clinical diagnosis using AECC criteria	Retrospective cohort study Conducted in 2 hospitals in Korea	Sensitivity 58% (33–80%) Specificity 80% (77–82%)	Not reported
Santiago *et al.* 2015 [6]	N = 218, all had sicca	Sjögren’s syndrome	ANA—indirect immunofluorescence	Minor salivary gland biopsy	Prospective cohort study at single hospital in Argentina	Sensitivity 84% (75–92%) Specificity 50% (42–59%)	Not reported
Ulvestad 2001 [7]	N = 446, unselected rheumatology patients, 4 of whom were diagnosed with SS	Rheumatology patient cohort	ANA—ELISA and indirect immunofluorescence	Preliminary European criteria	Retrospective cohort study; Norway	Indirect immunofluorescence sensitivity 73% (54–88%) Specificity 96% (93–97%) ELISA Sensitivity 63% (44–80%) Specificity 96% (77–82%)	
Ulvestad 2003 [8]	N = 407; unselected rheumatology patients, 73 of whom were diagnosed with SS	Rheumatology patient cohort	ANA—indirect immunofluorescence	Preliminary European criteria	Retrospective cohort study; Norway	Indirect immunofluorescence sensitivity 63% (51–73%) Specificity 76% (71–80%) And AURC 0.865	AURC: 0.865
Willems *et al.* 2018 [9]	N = 9856 Consecutive ANA tests; 63 later diagnosed with SS	Consecutive ANA tests; unselected	ANA—indirect immunofluorescence And FEIA—fluorenzyme immunoassay	ACR classification criteria	Retrospective cohort study; Belgium	Results reported as AURC: Indirect immunofluorescence 0.803 (0.799–0.892) FEIA 0.924 (0.876–0.971)	AURC:
Zafrir *et al.* 2013 [10]	N = 242; 67 healthy controls, 107 PBC; 20 scleroderma, 48 SS	Selected population of CTD and healthy controls in a single centre	ANA—indirect immunofluorescence	ACR classification criteria	Retrospective cohort study, Tel Aviv	Sensitivity 65% (49–78%) Specificity 97% (90–100%)	

The area under (a ROC) curve is a measure of the accuracy of a quantitative diagnostic test. A test with no better accuracy than chance has an AUC of 0.5, a test with perfect accuracy has an AUC of 1 [11]. AUC can be misleading as it gives equal weight to the full range of sensitivity and specificity values even though a limited range, or specific threshold, may be of practical interest [12].

AARD: antibody-associated rheumatic disease; AECC: American-European Consensus Classification; AURC: area under the curve.

Studies [5, 13, 14] (see Table 2) estimate the sensitivity of ENA as 89–92%; with a specificity of 71–77%. In a very small number of cases individuals can be ANA negative but Ro positive.

**Table 2.** Summary of evidence on diagnostic accuracy of extractable nuclear antigens (ENA) in SD

Study	Population	Diagnosis	Index Tests	Ref standard	Comments	Sensitivity % & Specificity% (95% CI) in CTD	AURC (95% CI)
Bentow *et al.* 2013 [13]	N=1079	Various AARDs including SD	Two tests: 7 test ENA panel (ELISA) 6 test NA panel (ELISA)	Unclear	Prospective cohort study; not specific to Sjögren’s	Sensitivity 92% (79–98%) Specificity 74% (71–77%)	0.97 (0.93–1.0)
Jeong *et al.* 2018 [5]	N=1115	Various AARDs including SD	9 test ENA panel (ELISA)	Expert clinical diagnosis using AECC	Retrospective cohort study	Sensitivity 90% (76–97%) Specificity 71% (68–73%)	0.97 (0.94–0.99)
Pi *et al.* 2012 [14]	N=329	Various AARDs including SD	Two tests: 6 test NA panel (ELISA) Multiplex bead based immunoassay (MPBI)	Physician diagnosed—criteria not specified	Retrospective cohort study	Sensitivity 89% (67–99%) Specificity 77% (74–79%)	0.94 (0.91–0.98)

The area under (a ROC) curve is a measure of the accuracy of a quantitative diagnostic test. A test with no better accuracy than chance has an AUC of 0.5, a test with perfect accuracy has an AUC of 1 [11]. AUC can be misleading as it gives equal weight to the full range of sensitivity and specificity values even though a limited range, or specific threshold, may be of practical interest [12].

AARD: antibody-associated rheumatic disease; AECC: American-European Consensus Classification; AURC: area under the curve.

None of the ‘novel’ autoantibodies out-perform anti-Ro antibody and are not recommended outside a research setting [15, 16].

### Recommendation

Do not measure ANA in the absence of clinical indicators of SD or other CTD (1, C) SOA 94.6%.

Use ANA as a screening antibody where there is a clinical suspicion of CTD (1, C) SOA 93.9%.

Measure ENA even if the ANA is negative if there is a high index of suspicion of SD (1, C) SOA 96.7%.

### 2a. In people suspected of SD, what is the diagnostic accuracy of salivary gland (SG) ultrasound scanning (USS)?

Multiple studies have confirmed the utility of SG USS in the diagnosis of SD in both adults and children [17–19], although it may not differentiate between SS and sarcoid or other connective tissue diseases (CTDs) [19] and does not form part of the classification criteria [20].

### Recommendation

USS of the salivary glands can provide useful additional information to support either the presence of or lack of evidence for SD (1, A) SOA 95.2%.

USS does not currently replace either antibody testing or histological analysis in adult SD classification criteria (1, A) SOA 96.4%.

### 2b. In people suspected of SD, what is the diagnostic accuracy of other imaging modalities?

Reviews of imaging modalities in SD [21, 22] have concluded that further studies are needed to establish the role of PET, CT and MRI. None of the imaging modalities are included in the most recent classification criteria.

### Recommendation

Overall, although they may provide useful supplementary information, we do not recommend additional imaging modalities over and above USS in the routine assessment of SD (1, C) SOA 97.3%

### 3a. In people suspected of SD, what is the diagnostic accuracy of major and minor salivary gland biopsy?

Minor (labial) SG biopsy—sensitivity 80–92%; specificity 88–97%—forms an essential part of the 2016 ACR/EULAR classification criteria when individuals are anti-Ro antibody negative [20]. Parotid gland biopsy has a sensitivity of 78% and specificity of 86% [23]. Complication rates of both are low overall, especially in those undergoing minimally invasive techniques [23].

### Recommendation

Consider a minor labial salivary gland biopsy to aid diagnosis in those with clinically suspected SD where the diagnosis cannot be made by clinical and serological features alone (1, A) SOA 98.2%

### 3b. In people suspected of SD, what is the diagnostic accuracy of lacrimal gland biopsy?

#### Recommendation

There is currently insufficient evidence to routinely recommend lacrimal gland biopsy in SD (1, C) SOA 98.2%

### 4a. In people with confirmed SD are there any measurable biomarkers that can predict development of lymphoma?

Certain factors consistently emerge as predictors of future lymphoma development in SD in adults and children [24, 25]:

- low C3/C4 with low C4 being the strongest predictor;
- clinical evidence of salivary gland enlargement;
- clinical evidence of lymphadenopathy;
- anti-Ro and/or La and RF;
- cryoglobulinaemia;
- conoclonal gammopathy; and
- high focus score [26].

### Recommendation

Individuals with SD should be offered further investigation early if they present with new salivary gland swelling or other symptoms that might suggest the development of lymphoma (1, A) SOA 98.75%.

Consider a minor labial salivary gland biopsy to provide additional prognostic data regarding lymphoma risk in both seronegative and seropositive individuals (2, C) SOA 92.7%.

### 4b. In people with confirmed SD, are there any measurable biomarkers that can predict disease progression or development of extra-glandular disease?

A number of features [27–29] are associated with a higher risk of progression to systemic extra-glandular disease:

- anti-Ro antibody positive;
- younger age of onset;
- ethnicity (Black/African-American);
- males; and
- RF positive.

### Recommendation

Baseline assessment of individuals with SD should include a thorough clinical and serological evaluation to inform the risk of development of extra-glandular features and disease progression (1, B) SOA 97.6%

### 5. In people with confirmed SD, what other investigations should routinely be undertaken to exclude common associated conditions; for example, coeliac or thyroid disease?

Co-morbidities are common, increase with age and body mass index (BMI) and commonly include osteoarthritis, gastro-oesophageal reflux and hypertension [30, 31]. There is a higher than expected incidence of positive tissue transglutaminase (TTG) IgA (TTG) and coeliac disease [32]. The commonest associated autoimmune liver condition is primary biliary cholangitis (PBC) affecting 4–9% of SD patients in studies of European and American populations [33–36]. Patients with SD are at higher risk of developing a monoclonal gammopathy (MGUS) with an odds ratio of 4.51 [37, 38]. The estimated prevalence of complete distal renal tubular acidosis (dRTA) is 5% and of incomplete 25% in an SD population [39, 40]. Muscle pain (myalgia) is common in SD but objective evidence of myositis is rare (0.45%) [41]. A systematic review and meta-analysis of vitamin D deficiency and severity of dry eye symptoms in SD [42] concluded that individuals with vitamin D deficiency had more severe dry eye disease. Compared with adults, children with jSD have more frequent neurologic and renal manifestations [43].

### Recommendation

Be aware of and consider screening for commonly associated conditions, as guided by age and/or clinical presentation (1, B) SOA 94.7%

We recommend that the following additional investigations are undertaken at baseline, and repeated as clinically indicated, to detect co-morbidities and associated autoimmune diseases:

- thyroid function;
- liver function tests (and anti-mitochondrial antibodies if indicated);
- TTG;
- immunoglobulins and serum electrophoresis;
- serum bicarbonate;
- creatine kinase;
- vitamin D levels (1, B) SOA 95.6%.

### 6. In people with SD who have sicca (dryness) symptoms of the eyes, what is the most clinically effective topical treatment?

Much of the evidence is based on studies looking at the dry eye population in general with very few looking exclusively at SD-related dry eye.

Cochrane and systematic reviews agree that artificial tears are safe and consistently improve ocular symptoms [44, 45]. They conclude that patients should be offered non-preserved or soft preserved artificial tears to avoid worsening of the dry eye disease due to the toxic, proinflammatory and detergent effects of the preservative. There is some evidence that combination formulations are more effective than single active ingredient artificial tears and that hyaluronic acid (HA) eye drops are superior to saline or non-HA based drops [45, 46]. The frequency of instillation of eye drops is important, with evidence suggesting that 2–3 hourly is optimum [47].

### Recommendation

Advise regular use of a preservative-free lubricating eye drop (e.g. 2–3 hourly) (1, A) SOA 94.4%

####  

##### Serum eye drops

Systematic reviews and meta-analysis of serum eyedrops for dry eye confirm improvement in patient-reported outcome measures, corneal staining and tear break-up time (TBUT) [48, 49]. In the UK, serum eye drops are only available via specialized centres in line with published NHS policy.

### Recommendation

Autologous or allogeneic serum eye drops may be offered to individuals with ongoing symptoms despite maximal management with conventional eye drops (1, A) SOA 91.9%

*NB: In the UK, serum eye drops are only available via specialised centres in line with published NHS policy.*

####  

##### Topical steroid eyedrops

A Cochrane review of topical corticosteroids for dry eye disease found a small-to-moderate improvement in symptoms and signs [50].

### Recommendation

Topical steroid eye drops, under ophthalmic supervision, may be offered short-term to individuals with ongoing persistent inflammation despite maximal management with conventional eye drops (1, A) SOA 94.9%

####  

##### Immunomodulating eye drops

###### Ciclosporin

A systematic literature review of the use of topical immunomodulatory drugs including ciclosporin found some evidence of improvement in corneal staining and TBUT [51]. Ciclosporin eye drops can be used off-label in children and adolescents from four years of age, based on the efficacy observed in keratoconjunctivitis [52], but there are no published studies in jSD.

### Recommendation

Topical ciclosporin eye drops, under ophthalmic supervision, may be indicated for patients with persistent surface inflammation despite maximal management with conventional eye drops (1, B) SOA 94.9%

#### Treatments for meibomian gland deficiency (MGD)

A systematic review of evidence-based treatments for MGD found all eight standard forms of treatment—including self-applied eyelid warming, thermal pulsation, IPL, MG probing, antibiotics, lipid containing eye drops and perfluorohexyloctane—were effective, although with variable clinical improvement [53].

### Recommendations

Advise a heated eyelid compress for at least 10 min daily (1, A) SOA 94.9%

Lipiflow, intense pulsed light therapy and meibomian gland probing are not currently NHS-funded as treatments within the UK. There is currently insufficient evidence to recommend their routine use. However, these procedures are safe with, in some cases, weak evidence of benefit in dry eye and individuals may decide to undergo these treatments in the private sector (2, C) SOA 84.5%.

#### Antibiotics for meibomian gland disease

A review of antibiotic treatment for dry eye disease with meibomian gland dysfunction or blepharitis [54] confirmed short-term improvements but felt there was insufficient evidence to recommend long-term use.

### Recommendation

Those with dry eye disease associated with meibomian gland dysfunction or blepharitis could be offered short-term treatment with oral or topical antibiotics with an anti-inflammatory action (2, B) SOA 92.3%

#### Lipid-containing eye drops

A systematic review of lipid-containing lubricants [55] confirmed short-lived symptomatic improvement.

### Recommendation

Individuals with dry eye disease associated with meibomian gland dysfunction or blepharitis could be advised to use lipid-containing eye drops or liposomal eye sprays as adjunctive treatment (2, C) SOA 90.2%

#### Punctal occlusion

A Cochrane review [56] concluded that the evidence of benefit was inconclusive although individual studies suggest that punctal plugs may improve symptoms. Expert opinion is that punctal plugs are suitable in some patients, but they may make corneal surface inflammation worse in certain situations.

### Recommendation

Punctal plugs are suitable in certain circumstances, but they may make corneal surface inflammation worse in certain situations. Careful patient selection is important (1, C) SOA 96.3%.

#### Androgen replacement therapy (ART)

A systematic review of Androgen replacement therapy (ART) in dry eye disease [57] concluded that despite short-term benefit there was insufficient evidence to recommend routine use.

### Recommendation

There is insufficient evidence to recommend androgen replacement therapy for dry eye disease (2, C) SOA 96.3%

### 7. In people with SD who have sicca (dryness) symptoms of the mouth, what is the most clinically effective topical treatment?

A Cochrane review [58] of topical treatments for dry mouth of any cause (including SD) found no strong evidence supporting one topical therapy over another.

A Cochrane review of non-pharmacological therapies for dry mouth [59] concluded that acupuncture was no different from placebo, there was insufficient evidence on the effect of an electrostimulation device and no difference between manual and powered toothbrushing symptoms.

### Recommendation

Suggest saliva substitutes for symptomatic relief of oral dryness (2, C) SOA 93.3%

### 8. In people with SD who have sicca (dryness) symptoms outside the eyes and mouth, what is the most clinically effective topical treatment?

#### Topical treatments for vaginal dryness

Vaginal dryness is a common symptom in patients with SD with low-to-moderate evidence of benefit from topical oestrogens [60] and non-hormonal vaginal moisturizers [61, 62].

Topical oestrogens are not recommended for use in children or adolescents.

### Recommendations

Consider advising topical non-hormonal vaginal moisturizers plus oestrogen creams/pessaries in peri- or post-menopausal women with significant vaginal dryness (2, C) SOA 97.5%

### 9a. In people with SD who have sicca (dryness) symptoms, what is the most clinically effective stimulatory treatment?

#### Stimulatory treatments for ocular sicca pilocarpine

There are no recent studies of pilocarpine in SD, but some good evidence of benefit from historical studies [63, 64, 65]. There is anecdotal evidence that starting with a low dose and titrating upwards over time reduces side effects.

### Recommendation

Consider a trial of pilocarpine (5 mg once daily increasing to 5 mg tds/qds) in those with significant ocular sicca symptoms with evidence of residual glandular function (1 A) SOA 95.3%

### What is the most clinically effective stimulatory treatment for oral dryness?

Two large RCTs [63, 65] confirmed significant improvement in oral dryness and salivary flow rates with pilocarpine but side effects were common.

### Recommendation

Consider a trial of pilocarpine (5 mg once daily increasing to 5 mg tds/qds) in those with significant oral sicca symptoms with evidence of residual glandular function (1, A) SOA 98.4%

### 9b. What is the clinical effectiveness of fluoride, xylitol, chlorhexidine, artificial saliva or diet in preventing the development or progression of dental caries and gum disease?

None of the published evidence is SD-specific and much is old. Most of the evidence relates to children and adolescents with little evidence in adults. There is evidence supporting fluoride toothpaste and xylitol in caries prevention but little evidence of benefit to chlorhexidene [66, 67, 68].

### Recommendation

Recommend regular brushing with fluoride toothpaste, proactive dental care and the use of xylitol-containing products as an alternative to sugar to prevent dental decay (2, C) SOA 95.6%

### 10a. In people with SD, what is the clinical effectiveness of treatments in comparison to each other or placebo for treating systemic disease?

Systemic (extra-glandular) features are seen in up to 70% of patients with SD and are severe in 15% [69]. Most commonly involved organs are joints, lungs, skin and peripheral nerves [70].

#### Conventional immunomodulatory drugs

##### Hydroxychloroquine

There is some weak evidence of benefit for hydroxychloroquine [71–78], but no new studies since the last guideline was published.

### Recommendation

In those with significant fatigue and systemic symptoms, consider a trial of hydroxychloroquine for 6–12 months (2, C) SOA 95.6%

#### Corticosteroids

There are case reports and small case series suggesting that corticosteroids help certain systemic features but no good evidence of benefit in general.

### Recommendation

Systemic steroids may be used short-term for specific indications but should not be offered routinely in the management of SD (2, C) SOA 97.7%

#### Conventional immunosuppressive drugs

Aside from hydroxychloroquine there have been several low-quality studies looking at other immunosuppressives [79–85]. All were small, mostly not RCT and most showed no benefit. The evidence for the use of immunosuppressive drugs other than hydroxychloroquine is poor and individual practice varies considerably. We would recommend that any treatment decisions are made on a case-by-case basis. Most of the conventional DMARDs can be used off-label in children from the age of two years with the exception of leflunomide which is not approved for use in people younger than 18 years.

### Recommendation

Conventional immunosuppressive drugs are not routinely recommended for use in SD outside of the treatment of specific systemic complications (2, C) SOA 94.7%

#### Treatment of systemic disease: biologic drugs

Biologics are not NICE-approved for SD. Of the few patients who do get biologics, this is usually either as part of a clinical trial or because they meet criteria for RA or another CTD (usually SLE).

With the exception of rituximab and belimumab, the evidence base for biologics in SD is unconvincing although a number of studies are currently underway looking at a range of biologic agents. Open label and phase II studies of belimumab alone and in combination with rituximab have demonstrated small improvements in ESSDAI score from baseline [86, 87]. The European guidelines have suggested belimumab as rescue therapy in those with severe systemic disease refractory to conventional immunosuppression and rituximab [88].

Two systematic reviews and a meta-analysis of rituximab treatment for SD [89, 90] found weak evidence of benefit but insufficient evidence to support routine use. It may have a role to play in patients with specific organ manifestations including ILD [91]. The North American guideline group recommend rituximab for systemic complications [92, 93] and the most recent European guidelines recommend rituximab for patients with severe, refractory systemic disease [88].

No studies have been performed in jSD, although rituximab may be used off-label for specific indications from 3–6 months, and belimumab from five years of age. Rituximab has been prescribed by paediatricians for selected jSD cases, with 40% of surveyed clinicians stating that they have used it for systemic manifestations and 9% for recurrent parotitis [94]. Rituximab has also been found beneficial in treating MALT lymphoma and neurological manifestations in children as per various case reports [95]. An NHS commissioning policy in 2016 declined to fund rituximab for patients with SD in England and Wales.

### Recommendation

Biologic drugs are not currently recommended for use in patients with SD outside of the treatment of specific systemic complications (2, C) SOA 93.5%

#### Treatment of systemic disease: miscellaneous

##### Intravenous immunoglobulins

There is anecdotal evidence supporting the use of intravenous immunoglobulin therapy in SD-associated sensorimotor and non-ataxic sensory neuropathy [96, 97] and refractory SD-associated myositis not responding to conventional treatment [41]. There is no evidence for its routine use in patients without significant systemic disease.

### Recommendation

Intravenous immunoglobulins are not routinely recommended for use in patients with SD outside of the treatment of specific systemic complications (2, C) SOA 96.9%

#### Colchicine

There are case reports describing successful treatment of SD-associated hypergammaglobulinaemic purpura [98], non-cryoglobulinaemic vasculitis [99], granulomatous panniculitis [100] and pericarditis [101] with colchicine. It is generally safe and well tolerated.

### Recommendation

Colchicine may be helpful in SD presenting with specific systemic complications (2, C) SOA 91.4%

### 10b. What treatments are beneficial for recurrent parotitis in jSD?

Recurrent, treatment-resistant parotitis can be a particular problem in jSD. A systematic review of the management of juvenile recurrent parotitis (not SD specific) [102] concluded that the available evidence was weak and difficult to interpret.

### Recommendation

Treatment of parotitis in jSD (once infection and stone disease excluded) could include the following escalating therapies: a short course of non-steroidal anti-inflammatory drugs or oral steroids combined with massage followed by washouts with saline or steroids. Consider anti-B-cell targeted therapies in selected, refractory cases (2, C) SOA 91%.

### 11. In people with SD, is early treatment of hypergammaglobulinaemia or systemic disease more effective than delayed treatment at slowing disease progression?

There is some evidence linking hypergammaglobulinaemia with disease progression and evidence that hydroxychloroquine reduces immunoglobulin levels, potentially influencing outcome [103,  104, 105].

### Recommendation

In SD with significant hypergammaglobulinemia, consider a trial of hydroxychloroquine for 6–12 months (2, C) SOA 94.2%

### 12. What are the recommended therapeutic options in individuals with SD overlapping with other rheumatic diseases; for example RA, SLE or scleroderma?

A number of conditions are commonly found in association with SD but the literature on management of these overlaps is scant and mostly based on anecdotal reports.

### Recommendation

In individuals with overlap CTDs, take all confirmed disease entities into account when planning investigation and management (2, C) SOA 96.3%

### 13. In people with SD, what is the clinical effectiveness of nutraceuticals in the management of the condition?

A 2021 review confirmed that local (topical) application of vitamin A was effective in reducing signs and symptoms of dry eye disease [106].

A Cochrane review [107] and a meta-analysis found weak evidence of symptomatic improvement in eye disease with omega-3 supplementation [108].

### Recommendation

Consider vitamin A-containing eye ointments (2, C) SOA 89.8%

Consider advising omega-3 supplementation in patients with SD (2, C) SOA 89.8%

### 14. For people with SD, what cognitive therapy or behavioural change interventions are an effective treatment for fatigue and joint pain?

Most studies are small and poor quality but aerobic exercise appears to be safe and effective [109].

### Recommendation

We recommend an individualized holistic review for those with fatigue focusing on activity management (for example planning, prioritizing, pacing), sleep quality and lifestyle (2, C) SOA 96.7%

### 15. In people with SD, what type and frequency of exercise is an effective treatment for fatigue?

Small studies of supervised resistance exercise, walking, cardiovascular exercise and Pilates have all shown improvements in well-being, fatigue and symptoms but with no change in ESSDAI [110–114].

### Recommendation

Exercise is safe and potentially beneficial for patients with SD (2, C) SOA 97.9%

### 16. For pregnant people with SD, both with and without anti-Ro and/or La antibodies, is hydroxychloroquine and/or low-dose aspirin effective in reducing fetal mortality and morbidity?

There is some evidence that hydroxychloroquine reduces the risk of congenital neonatal lupus (cNL) [115] and the prevalence of recurrent congenital heart block (CHB) [116]. Hydroxychloroquine and low-dose aspirin have both been shown to be safe in pregnancy [117].

### Recommendations

Recommend low-dose aspirin if high risk of pre-eclampsia or high-risk pregnancy in general (1, A) SOA 93.8%.

Consider hydroxychloroquine during pregnancy for those who are anti-Ro antibody positive on the basis of the risk reduction seen in the PATCH study (2, C) SOA 91.5%.

Offer hydroxychloroquine in subsequent pregnancies to those who have experienced congenital heart block in a previous pregnancy (1, B) SOA 96.7%.

### 17. For pregnant people with SD, with a foetus who has an incomplete heart block or hydropic changes, are fluorinated steroids and/or immunoglobulins effective in decreasing the likelihood of congenital heart block in the foetus?

Current UK practice varies but some units—for example, experts from Great Ormond Street Hospital—are treating with dexamethasone once CHB is detected. There is currently no international consensus on best practice.

### Recommendation

Refer urgently to specialist centre if CHB is detected for consideration of treatment with dexamethasone (2, C) SOA 98.9%

### 18. In people with SD, what is the most clinically effective long-term follow-up programme and how should this be personalized?

There is little evidence in the literature regarding optimum long-term follow-up of patients with SD.

### Recommendations

Consider follow-up within rheumatology for those with confirmed SD, particularly if there is evidence of systemic disease (2, C) SOA 91.9%

### 19. What age-tailored information, education and support do people with SD and their families and carers need and how can they access this?

Significant unmet needs have been identified within Europe for patients with SD and their families/carers [118] and efforts are underway to address this. Patients want tailored support from healthcare professionals including information provision, access to peer and professional support [119].

### Recommendation

Provide written information on the manifestations of SD and their management, direct individuals with SD to appropriate online resources and recommend that patients access local and national support groups, e.g. Sjögren’s UK (sjogrensuk.org), Sjögren’s Foundation (www.sjogrens.org), Versus Arthritis and NHS websites (2, C) SOA 97.1%

## Applicability and utility

The final guideline will be disseminated by publication in *Rheumatology* (Oxford) and will be freely available on the BSR website. It is recognized that constraints within the healthcare system may create challenges to widespread implementation of this guideline. For instance, many centres do not have access to minor salivary gland biopsy and not all have access to expert salivary gland USS. Access to certain treatments such as serum eye drops is limited by cost and availability and there are currently no immunomodulatory treatments licensed for use in SD. Most of the immunosuppressive drugs are used off licence for this indication. Biologics are not NICE approved for SD. Of the few patients who do get biologics this is usually either as part of a clinical trial or because they meet criteria for RA or another CTD (usually SLE).

## Research recommendations

There are significant unmet needs in the management of this patient cohort. Further research into pathogenetic mechanisms may facilitate the development of targeted treatments. Accurate stratification of patients into disease subgroups and collaborative studies is essential to provide large enough cohorts to demonstrate meaningful effects of interventions. There is a need to develop better measures of disease activity as the currently used parameters do not include fatigue and dryness, underestimate the disease burden and are not sensitive to change.

## Audit

A model audit tool is available via the BSR website and in Supplementary Data S2, available at *Rheumatology* online. We would also strongly recommend that new cases of SD are recorded in the NEIAA (New Early Inflammatory Arthritis Audit https://arthritisaudit.org.uk/) database to provide information on the incidence and demographics of the condition plus to collect evidence on diagnostic delays and route of referral.

## Conclusions

SD remains an under-recognized condition with significant unmet needs. Nonetheless we do feel that following these guidelines will provide a framework for health professionals to manage patients with SD effectively and proactively. There are studies underway investigating non-pharmacological treatments, novel biologic drugs and repurposing of existing conventional and biologic DMARDs and we would encourage teams to enrol patients into studies and national audits where possible.

## Supplementary Material

keae218_Supplementary_Data

## Data Availability

All data are available in the guideline and its supplementary material.
