# Supplementary material for: Executive summary: British Society for Rheumatology guideline on management of adult and juvenile onset Sjögren disease
Source: Rheumatology (Oxford). 2024 May 24;64(2):396–408. doi: 10.1093/rheumatology/keae218 (PMC12013822; doi:10.1093/rheumatology/keae218)
Supplement: keae218_Supplementary_Data [file keae218_supplementary_data.zip › Supplementary Data S1.docx]

**Summary of Search**

Restrictions in all databases were: Human, English language, and date range 1^st^ January 1990 – 1^st^ December 2022

Databases searched were:

PROQUEST (covers the following databases):

Consumer Health Database

Health & Medical Collection

Healthcare Administration Database

MEDLINE

Nursing & Allied Health Database

Psychology Database

Public Health Database

EBSCO (covers the following databases):

MEDLINE

MEDLINE Complete

CINAHL

Psychology and Behavioural Sciences Collection

OVID (covers the following databases):

AMED

Embase

Ovid Emcare

HMIC Health Management Information Consortium

Ovid Medline

COCHRANE LIBRARY
